# The learning curve of the distal radial access for coronary intervention

**DOI:** 10.1038/s41598-021-92742-7

**Published:** 2021-06-24

**Authors:** Ji Woong Roh, Yongcheol Kim, Oh-Hyun Lee, Eui Im, Deok-Kyu Cho, Donghoon Choi, Myung Ho Jeong

**Affiliations:** 1 Division of Cardiology, Department of Internal Medicine, Yonsei University College of Medicine and Cardiovascular Center, Yongin Severance Hospital, 363 Dongbaekjukjeon-daero, Giheung-gu, Yongin, 16995 Republic of Korea; 2 Department of Cardiology, Chonnam National University Hospital, Gwangju, Republic of Korea

**Keywords:** Cardiology, Interventional cardiology

## Abstract

Recently, coronary angiography (CAG) and percutaneous coronary intervention (PCI) via the distal radial access (DRA), are gaining attention owing to fewer complications. Despite the advantages of the DRA, there is difficulty to initiate this new vascular approach. The data from 1000 patients who underwent CAG and PCI via the DRA by a single experienced radial operator were retrospectively analyzed. The primary outcome was the success rate of the DRA per 100 cases. Moreover, the predictors of the failed DRA were analyzed. Overall, 952 (95.2%) of the total 1,000 patients underwent a successful DRA. After experiencing 200 cases, the DRA success rate was well maintained at > 94%, and there was no difference in success rate per 100 cases (*P*_trend_ = 0.216). The predictors of failure were female sex [odds ratio (OR) 1.84, 95% confidence interval (CI) 1.01–3.39, *P* = 0.049] and systolic blood pressure (SBP) of < 120 mmHg (OR 1.87, 95% CI 1.04–3.36, *P* = 0.036). For achieving a stable DRA with the success rate of > 94%, 200 procedures would be needed. Moreover, this new approach could fail in women and patients with low SBP.

Trial registration: https://cris.nih.go.kr/cris/index/index.do (Unique identifier: KCT0005349).

## Introduction

Recently, coronary angiography (CAG) and percutaneous coronary intervention (PCI) via the distal radial access (DRA), have shown potential benefits owing to fewer access-site complications including radial artery occlusion, and short hemostasis duration than the conventional radial approach^1,2^. Moreover, the feasibility of the DRA for patients with ischemic heart disease, including ST-elevation myocardial infarction (STEMI), has been demonstrated in several studies with minimal bleeding and fewer complications^3–6^. Despite the feasibility and potential benefits of the DRA, interventional cardiologists still find it difficult to initiate this new vascular approach because there is a lack of data regarding overcoming the learning curve, wherein the operator’s skills gradually improve with more experience, and choosing patients for initiating the DRA. Although success rate of DRA has been analyzed in several studies, there are no data on how many cases should be performed for achieving a consistently high success rate^3–5^.

Therefore, this study aims to investigate the learning curve for performing CAG and PCI via the DRA. Additionally, we intend to analyze the factors for the failed DRA.

## Methods

### Study population

The data from patients with suspected ischemic heart disease who underwent CAG and PCI via the DRA at a single center between November 2017 and November 2019 were retrospectively collected. The single experienced radial operator (Y.K.) attempted the DRA in patients with a well palpable pulse in or out the anatomical snuffbox area. The study protocol was approved by the institutional review board (IRB) of Chonnam National University hospital (CNUH) (approval number: CNUH-2020-231) and the requirement for informed consent was waived from the IRB of the CNUH because of the retrospective observational study design. All research was performed in accordance with relevant guidelines and regulation. This study was registered with Clinical Research Information Service (https://cris.nih.go.kr/cris/index/index.do, Unique identifier: KCT0005349).

### Process of the DRA

Puncture was performed using a 20-gauge two-piece needle with the through-and-through puncture technique or a 21-gauge open needle with the anterior wall puncture technique. After a successful puncture, a 0.025-inch straight wire or 0.018-inch hair wire was inserted, followed by the insertion of a 4-Fr to 7-Fr radial sheath (Radiofocus Introducer II; TERUMO Corporation, Tokyo, Japan or Prelude Radial; MERIT MEDICAL, UT, USA). The selection of the sheath size was made at the physician’s discretion. After successful sheath cannulation, a combination of 2.5 mg of verapamil, 0.2 mg of nitroglycerine, and 3000 units of unfractionated heparin diluted in 10 mL of saline solution was administered in all patients except those planned to undergo the ergonovine provocation test. Hemostasis was achieved using compression bandage with gauze.

### Study endpoints and definitions

The primary endpoint was the success rate of the DRA per 100 cases. Furthermore, the predictors of DRA failure were analyzed. Secondary endpoints were puncture attempts and median DRA time per 100 cases.

Puncture success refers to the case of blood pumping after puncture with a needle. The successful wiring with the sheath insertion after a puncture was called cannulation success and was also defined as DRA success. Puncture attempts was defined as the number of attempts to puncture with the needle at a completely different position until cannulation. DRA time was defined as the time interval between local anesthesia and complete sheath insertion. Forearm or distal radial artery occlusion was evaluated using palpation of pulse manually during hospitalization. Local numbness was also evaluated by the description of patients of a tingling sensation. Hematoma was divided to hand and forearm hematoma. Hand hematoma was classified as ≤ 5 cm diameter, 5–10 cm diameter, and > 10 cm diameter.

### Statistical analyses

Continuous variables were expressed as means with standard deviations or medians with interquartile ranges and were compared using the unpaired *t*-test. All categorical variables were represented as numbers with percentages and were analyzed using a χ^2^ test or Fisher’s exact test. Trends were analyzed using the Mantel–Haenszel test. The predictors of DRA failure were analyzed using the multivariable logistic regression model using factors with a *p* value of < 0.1 in the univariate model. Statistical analyses were conducted using R version 3.5.0 (The R Foundation for Statistical Computing, Vienna, Austria) and SPSS 25.0 for Windows (SPSS-PC, Chicago, IL, USA).

## Results

Overall, 1000 consecutive patients who underwent CAG and PCI via the DRA were analyzed in this study. The mean age was 66.3 ± 10.9 years, and 733 patients (73.3%) were men. Among 1,000 patients, 372 patients were performed PCI via the DRA and had a success rate of 98.4% (366/372). The details of PCI via the DRA were described in supplementary Table 1.

### Outcomes

**Figure 1:**
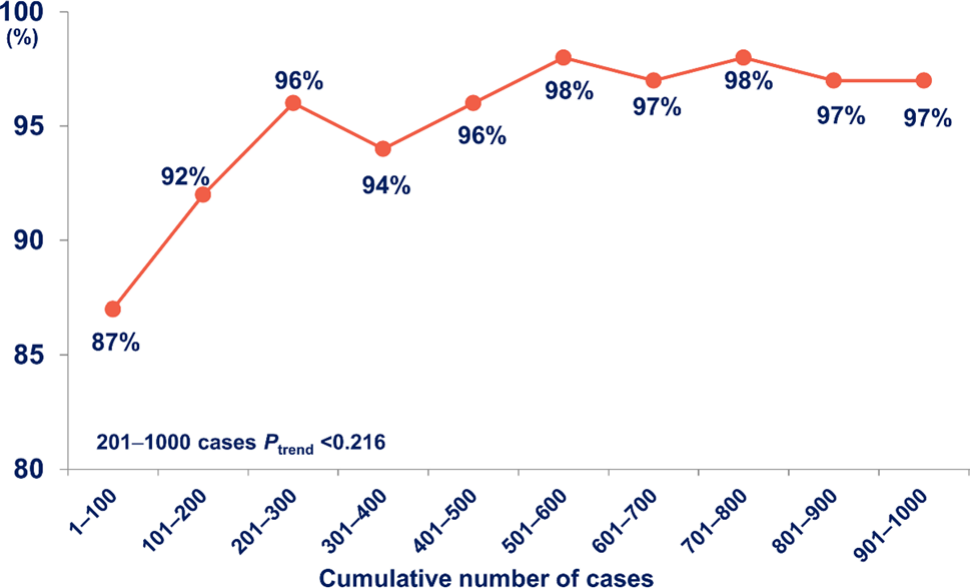
Temporal trend in success rate of the distal radial access showing the stable success rate (> 94%) after 200 cases.

**Figure 2:**
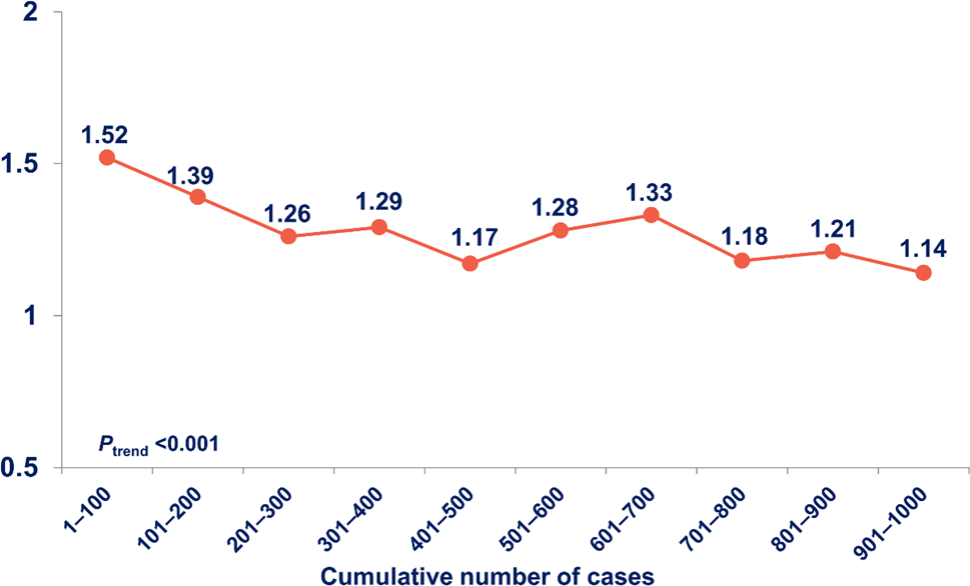
Temporal trend in puncture attempts of the distal radial access showing significantly decreasing from 1.52 in 1–100 patients to 1.14 in 901–1000 patients.

**Figure 3:**
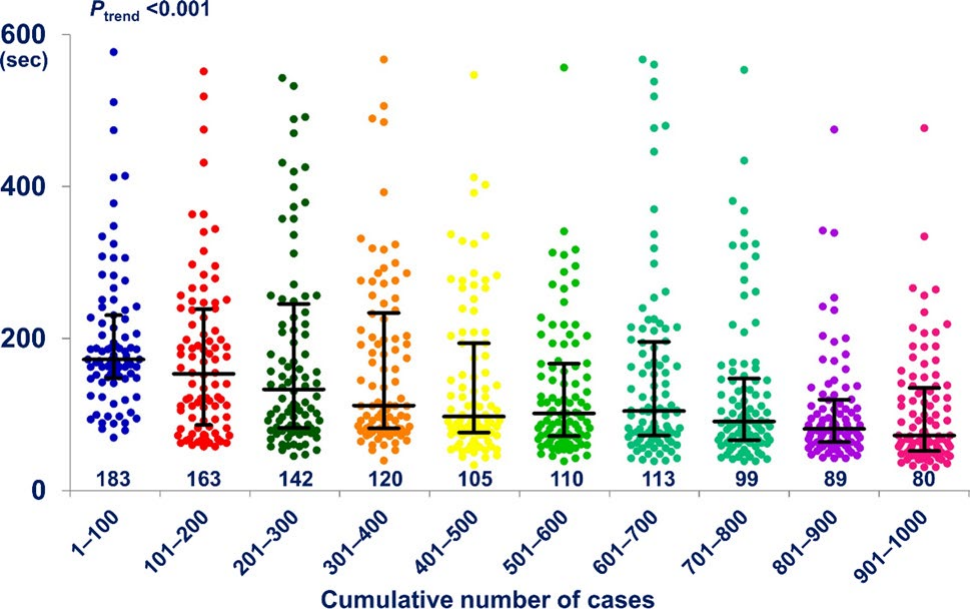
Trend analysis of median distal radial access time showing significantly decreasing from 183 s in 1–100 patients to 80 s in 901–1000 patients.

Overall, 952 (95.2%) of the 1,000 patients underwent a successful DRA (Table 1). Among the 48 (4.8%) patients with a failed DRA, 27 (2.7%) patients had failed wiring and cannulation, and 21 (2.1%) patients had failed puncture. Trend analysis showed that the success rate gradually increased (*P*_trend_ < 0.001). After experience with 200 cases, the success rate was well maintained at > 94%, and there was no difference in the success rate per 100 cases (*P*_trend_ = 0.216) (Fig. 1). All cases with the failed DRA succeeded by switching to the conventional radial approach and none of the cases were switched to the femoral approach. The average puncture attempts were 1.27 ± 0.61 for all DRA success patients (Table 1). The puncture attempts decreased gradually from 1.52 to 1.14 (*P*_trend_ < 0.001) (Fig. 2). The median DRA time was 117.5 [81.0–203.3] s. Moreover, DRA time decreased gradually when analyzing the trend per 100 cases (P_trend_ < 0.001) (Fig. 3).

**Table 1:** Distal radial access characteristics and complications.

Total patients	Total (N = 1000)
DRA success	952 (95.2%)
Failed DRA	48 (4.8%)
Crossover to the conventional radial approach	48 (100%)
Ipsilateral	40 (83.3%)
Contralateral	8 (16.7%)
Crossover to femoral approach	0 (0%)

DRA details	N = 952
Puncture attempts	1.27 ± 0.61
DRA time (s)	117.5 [81.0–203.3]
Left DRA	900 (94.5%)
Hemostasis duration	
Total patients (mins)	153.8 ± 62.1
CAG patients (n = 580) (mins)	144.6 ± 91.3
PCI patients (n = 372) (mins)	217.3 ± 121.3
PCI success rate (n = 372)	366 (98.4%)

Access-site complications	N = 952
Forearm RA occlusion	0 (0%)
Distal RA occlusion	0 (0%)
Local numbness	2 (0.2%)
Hand hematoma	29 (3.0%)
≤ 5 cm diameter	22 (2.3%)
5–10 cm diameter	4 (0.4%)
> 10 cm diameter	3 (0.3%)
Forearm hematoma	0 (0%)

Values are presented as mean ± standard deviation, numbers (%), or median [interquartile range].

*DRA* Distal radial access, *CAG* Coronary artery angiography, *PCI* Percutaneous coronary intervention, *RA* Radial artery.

### Hemostasis duration and access-site complications

Among the DRA success group, the left DRA was 94.5% (900/952). For hemostasis duration, it was approximately 2 h (144.6 ± 91.3 min) for CAG (n = 580) and 3.5 h (217.3 ± 121.3 min) for PCI (n = 372). For access-site complications, there was no forearm and distal radial artery occlusion during hospitalization. Puncture-related local numbness was observed in two cases (0.2%), and local hematoma occurred in 29 (3.0%) cases without major bleeding complications requiring surgery or transfusion (Table 1).

### Factors associated with the failed DRA

Baseline clinical characteristics of our study population were divided into two groups: the DRA success (n = 952) and the failed DRA (n = 48) groups. The failed group had less hypertension and lower proportion of men than the success group (Table 2). The multivariable analysis revealed female sex [odds ratio (OR) 1.84, 95% confidential interval (CI) 1.01–3.39, *P* = 0.049] and systolic blood pressure (SBP) < 120 mmHg (OR 1.87, 95% CI 1.04–3.36, *P* = 0.036) as independent predictors of the failed DRA (Table 3).

**Table 2:** Baseline clinical characteristics of the study population.

Patients	Total (N = 1000)	DRA success (N = 952)	DRA failed (N = 48)	*p* value
**Demographics**				
Age, years	66.3 ± 10.9	66.3 ± 11.0	65.9 ± 11.2	0.630
Male	733 (73.3%)	703 (73.8%)	30 (62.5%)	0.083
Height	163.2 ± 8.9	163.6 ± 8.8	161.9 ± 10.6	0.271
Weight	66.0 ± 11.3	66.1 ± 11.4	65.2 ± 12.8	0.632
Body mass index (kg/m^2^)	24.6 ± 3.2	24.6 ± 3.15	24.8 ± 3.46	0.505
Body mass index > 25	435 (43.5%)	408 (42.9%)	27 (56.3%)	0.068
**Vital signs**				
SBP (mmHg)	126.4 ± 21.7	126.5 ± 21.6	124.5 ± 23.5	0.302
DBP (mmHg)	73.7 ± 13.9	73.9 ± 13.9	71.4 ± 13.9	0.845
Heart rate (bpm)	75.1 ± 13.1	75.1 ± 13.1	75.3 ± 14.4	0.614
**Risk factors**				
Hypertension	703 (70.3%)	676 (71.0%)	27 (56.3%)	0.026
Diabetes mellitus	329 (32.9%)	316 (33.2%)	13 (27.1%)	0.372
Current smoking	203 (20.3%)	194 (20.4%)	9 (18.8%)	0.774
CKD (eGFR < 60 mL/min/1.73 m^2^)	150 (15.0%)	143 (15.0%)	7 (14.6%)	0.932
Hemodialysis	36 (3.6%)	36 (3.8%)	0 (0%)	0.169
**Laboratory findings**				
Hemoglobin (g/dL)	13.4 ± 2.0	13.4 ± 2.0	13.4 ± 1.6	0.164
Platelets, 10^3^/mm^3^	228 ± 66	227 ± 66	233 ± 67	0.700
PT-INR	1.01 ± 0.14	1.01 ± 0.15	0.99 ± 0.06	0.069
**Reasons for CAG**				0.132
CCS	417 (41.7%)	402 (42.2%)	15 (31.3%)	
ACS	583 (58.3%)	550 (57.8%)	33 (68.8%)	
STEMI	65 (6.5%)	63 (6.6%)	2 (4.2%)	0.501
Ejection fraction	62.1 ± 24.9	61.9 ± 25.5	65.3 ± 8.9	0.377
**Periprocedural anti-thrombotic medication**				
Aspirin	976 (97.6%)	929 (97.6%)	47 (97.9%)	0.774
P2Y_12_ inhibitor	974 (97.4%)	927 (97.4%)	47 (97.9%)	0.556
Clopidogrel	820 (82.0%)	778 (81.7%)	42 (87.6%)	0.309
Ticagrelor	80 (8.0%)	76 (8.0%)	4 (8.3%)	0.930
Prasugrel	74 (7.4%)	73 (7.7%)	1 (2.1%)	0.369
Oral anticoagulation	62 (6.2%)	60 (6.3%)	2 (4.2%)	0.736

Values are presented as mean ± standard deviation, numbers (%).

*SBP* Systolic blood pressure, *DBP* Diastolic blood pressure, *CKD* Chronic kidney disease, *eGFR* Estimated glomerular filtration rate, *PT-INR* Prothrombin time-international normalized ratio, *CAG* Coronary angiography, *CCS* Chronic coronary syndrome, *ACS* Acute coronary syndrome, *STEMI* ST-elevation myocardial infarction.

**Table 3:** Predictors of distal radial access failure.

Overall patients (N = 1000)	Univariate analysis		Multivariable analysis	
	OR (95% CI)	*p* value	OR (95% CI)	*p* value
Age < 65 years	1.12 (0.63–2.01)	0.695		
Age > 80 years	0.84 (0.30–2.39)	0.743		
Female	1.69 (0.93–3.09)	0.086	1.84 (1.01–3.39)	0.049
SBP < 120 mmHg	1.80 (1.01–3.23)	0.047	1.87 (1.04–3.36)	0.036
Heart rate > 80 bpm	0.90 (0.45–1.79)	0.769		
Body mass index > 25 kg/m^2^	1.80 (0.94–3.46)	0.075	1.88 (0.98–3.62)	0.058
Current smoker	0.89 (0.43–1.88)	0.774		
History of diabetes mellitus	0.74 (0.39–1.43)	0.373		
CKD (eGFR < 60 mL/min/1.73 m^2^)	0.97 (0.42–2.19)	0.932		
Hemoglobin < 12.0 g/dL	0.71 (0.33–1.54)	0.388		
Acute coronary syndrome	1.61 (0.86–3.00)	0.136		

*SBP* Systolic blood pressure, *CKD* Chronic kidney disease, *eGFR* Estimated glomerular filtration rate.

## Discussion

**Figure 4:**
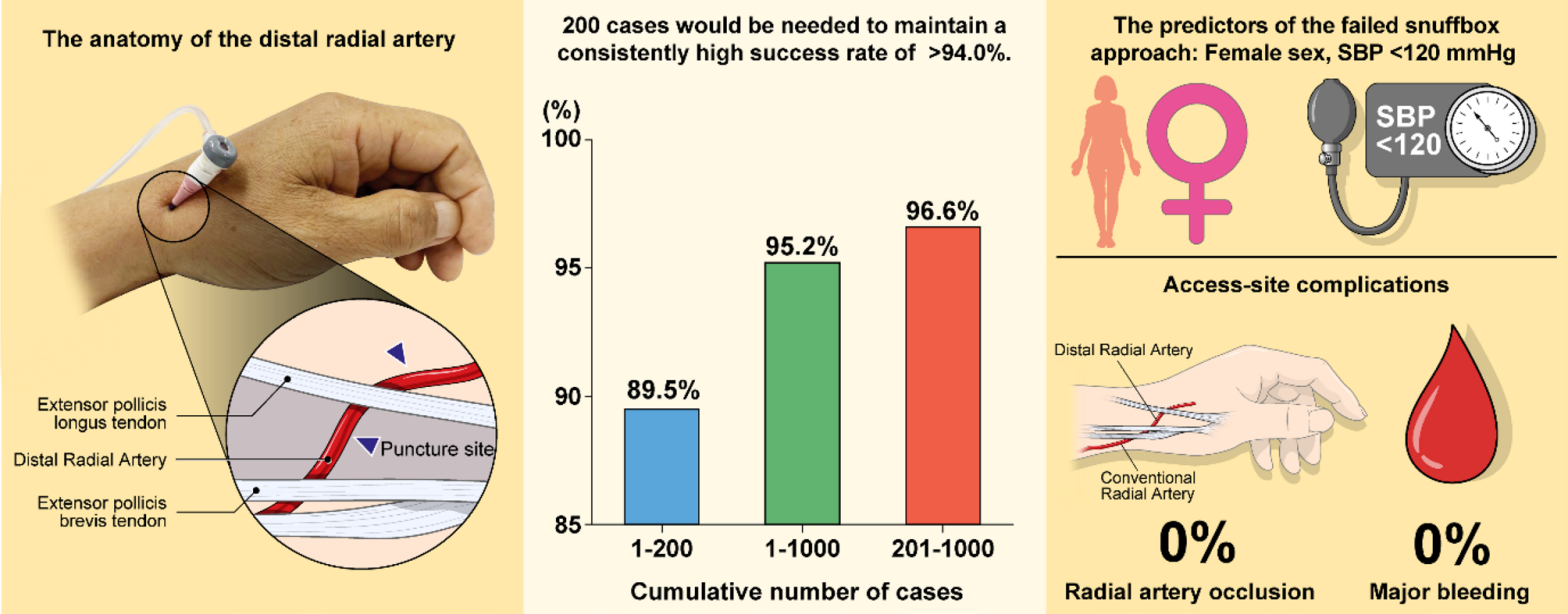
Summary of study regarding the learning curve of the distal radial access (DRA) for coronary intervention. (Left panel) Distal radial artery puncture site in the anatomical snuffbox located between tendons of the extensor pollicis longus and the extensor pollis brevis. (Central panel) Success rate of DRA showing 95.2% in a total of 1000 study population, 89.5% in 1–200 patients, and 96.6% in 201–1000 patients. (Right panel) Predictors of the failed DRA and access-site complications.

In our learning curve study of the DRA, we found that 200 cases of the DRA were required to maintain a consistently high success rate of > 94.0%. Moreover, puncture attempts and DRA time decreased gradually as the operator’s experience with the new vascular approach increased. Female sex and SBP < 120 mmHg were significant and independent predictors of the failed DRA (Fig. 4). To our knowledge, this is the first study to report the learning curve of the DRA for coronary intervention.

The concept of a learning curve for vascular intervention has been observed for many procedures, including the trans-radial intervention, although no studies have attempted to quantify this relationship for the DRA^7–9^. Thus, technical challenges may discourage operators from starting a new vascular approach despite the potential benefits and feasibility of the DRA over the conventional radial approach^3–5^. In our study, the success rate of the learning curve gradually improved from the initial starting point with a stable success trend after 200 cases. The result that the success rate improves as the operator’s experience increases suggests that this new puncture technique also has a learning curve like the conventional radial approach. Interestingly, puncture attempts and DRA time improved gradually over time as well. Therefore, operators who perform the DRA for the first time would have to perform approximately 200 procedures to achieve a consistently high success rate for the DRA.

Some data on the success rate of the DRA have been reported. Since Kiemeneij first reported the success rate of the DRA as 89.0% (62/70) in 2017, various follow-up studies have reported the success rate of the DRA from 88.0% (132/180) to 100% (54/54)^3–5,10–13^. Recently, a success rate of 92.8% was reported in the setting of STEMI (128/138)^6^. There was no random study conducted for investigating the success rates between distal and conventional radial approaches, but the smaller diameter of the distal radial artery, when compared to the conventional radial artery, suggested that it takes more time to overcome the learning curve of the DRA^14^. Additionally, there are no reports of failure factors of the DRA despite the importance of choosing appropriate patients to shorten the learning curve. Our study showed that female sex and SBP < 120 mmHg were significant factors associated with the failed DRA. This could be because women have a smaller distal radial artery diameter than men: 2.40 ± 0.53 mm versus 2.65 ± 0.46 mm (*P* < 0.016) on angiography, and 2.5 ± 0.5 mm versus 2.6 ± 0.5 mm on ultrasonography (*P* < 0.08), respectively^13,14^. For SBP < 120 mmHg, there is no related study, but it can be assumed that there would be difficulties to perform puncture if the pulse was weakly palpable owing to low blood pressure at the point of the distal radial artery. It is expected that it could be easier to overcome the learning curve if operators who want to perform DRA for the first time select a male patient with a high SBP.

There are several interesting results in our study. Firstly, in all patients in whom the DRA failed, the procedure was converted to the conventional radial approach: 83.3% in the ipsilateral and 16.7% in the contralateral access-site. It is possible to change quickly and easily to the ipsilateral radial, even if the operator fails the DRA. Secondly, access-site complications, including local numbness and major hematoma, were rare, and there was no forearm and distal radial artery occlusion. In a systemic review and meta-analysis for the DRA, the overall rate of complications was 2.4% in a total of 4,209 cases, and the radial artery occlusion was only 1.7% among the 2,003 cases of the DRA^15^. Therefore, this study observed the potential benefits of the DRA with less access-site complications, including forearm and distal radial artery occlusions as described in previous study.

The limitations of this study are as follows. First, this study has the inherent limitation owing to its retrospective nature. Second, since it involves data from a single operator, it is difficult to generalize our findings for all operators performing CAG and PCI. In contrast, our study can confirm the consistent improving trend of the DRA success rate, puncture attempts, and time in a large sample of 1,000 patients because it was performed by a single operator. Third, the occurrence of both forearm and distal radial artery occlusion was investigated during only hospitalization and was not evaluated using ultrasonography, although a reduction in the risk of radial artery occlusion is a potential benefit of the DRA.

## Conclusions

Two hundred cases of DRA for CAG and PCI were required to be performed to overcome the learning curve with consistently high success rates, and the puncture attempts and DRA time also gradually improved as the number of cases increased. Moreover, female sex and SBP < 120 mmHg were factors associated with the failed DRA. Regarding access-site complications, low incidence of minor hematoma was observed without forearm and distal radial artery occlusion. Prospective studies are needed to further confirm the learning curve period and predictors of the failed DRA.

## Supplementary Information

Supplementary Information.
