# Supplementary material for: The learning curve of the distal radial access for coronary intervention
Source: Sci Rep. 2021 Jun 24;11:13217. doi: 10.1038/s41598-021-92742-7 (PMC8225842; doi:10.1038/s41598-021-92742-7)
Supplement: Supplementary file 1 — Supplementary Information. [file 41598_2021_92742_MOESM1_ESM.docx]

Supplmentary Table 1. Clinical characteristics of the PCI (N=372)

| **Characteristic** | **Value** |
| --- | --- |
| **Reason for the failed PCI (N=6)** |  |
| Chronic total occlusion lesion | 4 (66.7%) |
| Severe calcified lesion | 2 (33.3%) |
| **Procedure details for successful PCI (N=366)** |  |
| PCI sheath size |  |
| 5-Fr sheath | 26 (7.1%) |
| 6-Fr sheath | 338 (92.3%) |
| 7-Fr sheath | 2 (0.5%) |
| Stent implantation | 337 (92.1%) |
| Case ≥ two stents implantation | 76 (20.8%) |
| Multivessel PCI | 37 (10.1%) |
| Treated lesion (n=457) |  |
| Left main coronary artery | 14 (3.1%) |
| Left anterior descending artery | 230 (50.3%) |
| Left circumflex artery | 99 (21.7%) |
| Right coronary artery | 114 (24.9%) |
| ACC/AHA type B2/C lesion | 368 (80.5%) |
| Type A | 31 (6.8%) |
| Type B1 | 58 (12.7%) |
| Type B2 | 117 (25.6%) |
| Type C | 251 (54.9%) |
| Bifurcation lesion | 155 (33.9%) |
| Chronic total occlusion | 12 (2.6%) |
| Intravascular modality-guided PCI | 98 (21.4%) |
| Fractional flow reserve guidance | 28 (6.1%) |
| Optical coherence tomography guidance | 44 (9.6%) |
| Intravascular ultrasound guidance | 26 (5.7%) |
| Total procedure time, min | 37.8 ± 15.2 |
| Total contrast volume, mL | 137.8 ± 48.5 |
| **Access-site complications** | **N = 372** |
| Forearm RA occlusion | 0 (0) |
| Distal RA occlusion | 0 (0) |
| Hand hematoma | 14 (3.8%) |
| ≤ 5 cm diameter | 10 (2.7%) |
| 5 – 10 cm diameter | 1 (0.3%) |
| > 10 cm diameter | 3 (0.8%) |
| Forearm hematoma | 0 (0%) |

Values are presented as mean ± standard deviation, numbers (%). DRA, Distal radial access; PCI, percutaneous coronary intervention; CAG, coronary artery angiography; RA, radial artery.
